# Poly[di-μ_5_-adipato-κ^4^ *O*:*O*′:*O*′′:*O*′′′,*O*′′′-μ_4_-adipato-κ^4^ *O*:*O*′:*O*′′:*O*′′′-bis­[2-phenyl-1*H*-1,3,7,8-tetra­azacyclo­penta­[*l*]phenanthrene-κ^2^ *N* ^7^,*N* ^8^]tricobalt(II)]

**DOI:** 10.1107/S1600536808010738

**Published:** 2008-04-23

**Authors:** Mao-Liang Xu, Rui Zhou, Ge-Yang Wang, Seik Weng Ng

**Affiliations:** a Xi’an Modern Chemistry Research Institute, Xi’an 710065, People’s Republic of China; b Department of Chemistry, University of Malaya, 50603 Kuala Lumpur, Malaysia

## Abstract

In the title polymer, [Co_3_(C_6_H_8_O_4_)_3_(C_19_H_12_N_4_)_2_]_*n*_, two adipate dianions (C_6_H_8_O_4_^2−^) occupy general positions and two are situated on different inversion centres. The two on general positions bind through their four O atoms to five 2-phenyl-1*H*-1,3,7,8-tetra­azacyclo­penta­[*l*]phenanthrene-chelated Co^II^ ions, whereas the two on special positions bind to only four. Of the three Co atoms, two are chelated by *N*-heterocycles; the third is bonded to six O atoms. The bonding mode of the dianion gives rise to a three-dimensional network structure; the network is further consolidated by N—H⋯O hydrogen bonds.

## Related literature

There are several studies of 4-(1*H*-1,3,7,8-tetra­azacyclo­penta­[*l*]phenanthren-2-yl)phenol transition metal dicarboxyl­ate compounds. See, for example: Che (2006*a* ▶,*b* ▶); Che *et al.* (2006 ▶); Wang, Yu, Meng *et al.* (2006 ▶); Wang, Yu, Zhao *et al.* (2006 ▶).
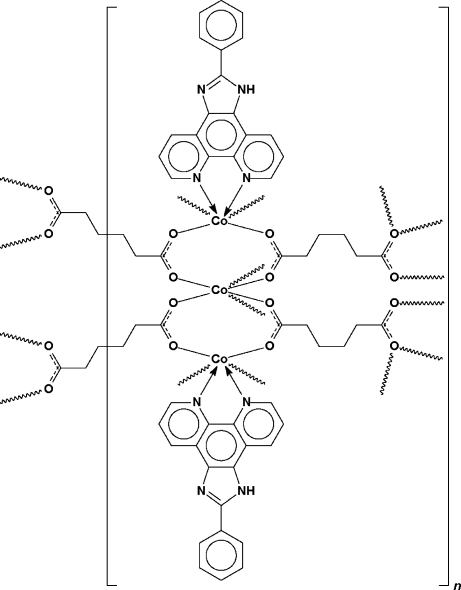


## Experimental

### 

#### Crystal data

[Co_3_(C_6_H_8_O_4_)_3_(C_19_H_12_N_4_)_2_]
*M* *_r_* = 1201.81
Triclinic, 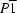

*a* = 8.150 (4) Å
*b* = 15.896 (8) Å
*c* = 20.474 (9) Å
α = 69.48 (2)°
β = 83.23 (2)°
γ = 87.34 (2)°
*V* = 2467 (2) Å^3^
*Z* = 2
Mo *K*α radiation
μ = 1.07 mm^−1^
*T* = 295 (2) K
0.11 × 0.10 × 0.09 mm

#### Data collection

Rigaku R-AXIS RAPID diffractometer
Absorption correction: multi-scan (*ABSCOR*; Higashi, 1995 ▶) *T*_min_ = 0.632, *T*_max_ = 0.910
23585 measured reflections
10947 independent reflections
5288 reflections with *I* > 2σ(*I*)
*R*_int_ = 0.094

#### Refinement

*R*[*F* ^2^ > 2σ(*F* ^2^)] = 0.067
*wR*(*F* ^2^) = 0.178
*S* = 1.02
10947 reflections
712 parameters
H-atom parameters constrained
Δρ_max_ = 0.64 e Å^−3^
Δρ_min_ = −0.59 e Å^−3^

### 

Data collection: *RAPID-AUTO* (Rigaku, 1998 ▶); cell refinement: *RAPID-AUTO*; data reduction: *CrystalStructure* (Rigaku/MSC, 2002 ▶); program(s) used to solve structure: *SHELXS97* (Sheldrick, 2008 ▶); program(s) used to refine structure: *SHELXL97* (Sheldrick, 2008 ▶); molecular graphics: *X-SEED* (Barbour, 2001 ▶); software used to prepare material for publication: *publCIF* (Westrip, 2008 ▶).

## Supplementary Material

Crystal structure: contains datablocks global, I. DOI: 10.1107/S1600536808010738/bh2161sup1.cif

Structure factors: contains datablocks I. DOI: 10.1107/S1600536808010738/bh2161Isup2.hkl

Additional supplementary materials:  crystallographic information; 3D view; checkCIF report

## Figures and Tables

**Table 1:** Selected bond lengths (Å)

Co1—O1	2.016 (5)
Co1—O3	2.022 (4)
Co1—O5^i^	2.156 (4)
Co1—O6^i^	2.274 (5)
Co1—N1	2.098 (5)
Co1—N2	2.163 (4)
Co2—O2	2.088 (4)
Co2—O4	2.040 (4)
Co2—O7	2.061 (4)
Co2—O9	2.039 (4)
Co2—O5^i^	2.167 (4)
Co2—O11^ii^	2.176 (4)
Co3—O8	2.030 (4)
Co3—O10	2.027 (4)
Co3—O11^ii^	2.159 (4)
Co3—O12^ii^	2.264 (4)
Co3—N5	2.097 (5)
Co3—N6	2.166 (4)

Symmetry codes: (i) 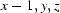; (ii) 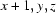.

**Table 2:** Hydrogen-bond geometry (Å, °)

*D*—H⋯*A*	*D*—H	H⋯*A*	*D*⋯*A*	*D*—H⋯*A*
N3—H3*N*⋯O6^iii^	0.86	2.05	2.785 (6)	143
N7—H7*N*⋯O12^iv^	0.86	2.05	2.795 (6)	144

Symmetry codes: (iii) 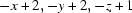; (iv) 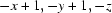.

**Fig. 1.**
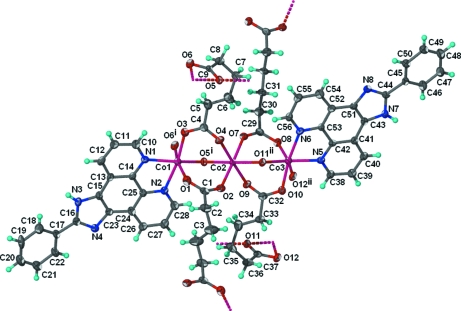
Thermal ellipsoid plot of a portion of polymeric Co3(C19H12N4)2(C6H8O4)3; displacement ellipsoids are drawn at the 50% probability level, and H atoms as spheres of arbitrary radii. Symmetry codes are given in Table 1.

## supplementary crystallographic information

### Comment

The 4-(1*H*-1,3,7,8-tetraazacyclopenta[*l*]phenanthren-2-yl)phenol *N*-heterocycle furnishes adducts with metal dicarboxylates,*i.e*. the manganese phthalate adduct (Wang *et al.*, 2006*b*), manganese isophthalate adduct (Che, 2006*b*), manganese terephthalate adduct (Che *et al.*, 2006), the zinc naphthalene-1,4-dicarboxylate adduct (Wang, Yu, Meng *et al.*, 2006), and the zinc terephthalate adduct (Che, 2006*a*).

### Experimental

Cobalt(II) acetate (0.1 mmol), adipic acid, 4-(1*H*-1,3,7,8-tetraazacyclopenta[*l*]phenanthren-2-yl)phenol (0.1 mmol) and water (12 ml) were heated in a 23 ml, Teflon-lined, stainless-steel Parr bomb at 408 K for 2 days. Crystals were obtained in 40% yield.

### Refinement

The carbon- and nitrogen-bound H atoms were placed in calculated positions [C—H 0.93 (aromatic CH) or 0.97 Å (methylene CH_2_); N—H 0.86 Å, and *U*_iso_(H) = 1.2*U*_eq_(carrier C,*N*)], and were included in the refinement in the riding-model approximation.

### Crystal data

**Table:**

[Co_3_(C_6_H_8_O_4_)_3_(C_19_H_12_N_4_)_2_]	*Z* = 2
*M**_r_* = 1201.81	*F*_000_ = 1234
Triclinic, *P*1	*D*_x_ = 1.618 Mg m^−^^3^
Hall symbol: -P 1	Mo *K*α radiation λ = 0.71073 Å
*a* = 8.150 (4) Å	Cell parameters from 12081 reflections
*b* = 15.896 (8) Å	θ = 3.0–27.5º
*c* = 20.474 (9) Å	µ = 1.07 mm^−^^1^
α = 69.48 (2)º	*T* = 295 (2) K
β = 83.23 (2)º	Block, brown
γ = 87.34 (2)º	0.11 × 0.10 × 0.09 mm
*V* = 2467 (2) Å^3^	

### Data collection

**Table:**

Rigaku R-AXIS RAPID diffractometer	10947 independent reflections
Radiation source: fine-focus sealed tube	5288 reflections with *I* > 2σ(*I*)
Monochromator: graphite	*R*_int_ = 0.094
Detector resolution: 10 pixels mm^-1^	θ_max_ = 27.5º
*T* = 295(2) K	θ_min_ = 3.0º
ω scans	*h* = −10→10
Absorption correction: multi-scan(ABSCOR; Higashi, 1995)	*k* = −20→20
*T*_min_ = 0.632, *T*_max_ = 0.910	*l* = −26→26
23585 measured reflections	

### Refinement

**Table:**

Refinement on *F*^2^	Secondary atom site location: difference Fourier map
Least-squares matrix: full	Hydrogen site location: inferred from neighbouring sites
*R*[*F*^2^ > 2σ(*F*^2^)] = 0.067	H-atom parameters constrained
*wR*(*F*^2^) = 0.179	*w* = 1/[σ^2^(*F*_o_^2^) + (0.0677*P*)^2^ + 0.2367*P*] where *P* = (*F*_o_^2^ + 2*F*_c_^2^)/3
*S* = 1.02	(Δ/σ)_max_ = 0.001
10947 reflections	Δρ_max_ = 0.64 e Å^−^^3^
712 parameters	Δρ_min_ = −0.59 e Å^−^^3^
Primary atom site location: structure-invariant direct methods	Extinction correction: none

### Fractional atomic coordinates and isotropic or equivalent isotropic displacement parameters (Å^2^)

**Table:**

	*x*	*y*	*z*	*U*_iso_*/*U*_eq_	
Co1	0.65991 (10)	0.83605 (6)	0.38528 (4)	0.0322 (2)	
Co2	0.73442 (10)	0.73830 (5)	0.25912 (4)	0.02684 (19)	
Co3	0.81551 (9)	0.64935 (5)	0.12644 (4)	0.0297 (2)	
O1	0.7209 (5)	0.7077 (3)	0.4380 (2)	0.0448 (11)	
O2	0.6863 (5)	0.6431 (3)	0.35964 (19)	0.0392 (11)	
O3	0.8855 (5)	0.8859 (3)	0.3400 (2)	0.0440 (11)	
O4	0.9352 (5)	0.7868 (3)	0.2850 (2)	0.0405 (11)	
O5	1.5779 (4)	0.8357 (3)	0.28902 (17)	0.0300 (9)	
O6	1.5515 (5)	0.9617 (3)	0.3092 (2)	0.0407 (11)	
O7	0.7836 (5)	0.8353 (3)	0.16135 (18)	0.0381 (10)	
O8	0.7492 (5)	0.7790 (3)	0.07753 (19)	0.0384 (10)	
O9	0.5385 (5)	0.6889 (3)	0.2308 (2)	0.0369 (10)	
O10	0.5899 (5)	0.5970 (3)	0.1704 (2)	0.0387 (10)	
O11	−0.1084 (4)	0.6433 (3)	0.22552 (18)	0.0308 (9)	
O12	−0.0707 (5)	0.5232 (3)	0.1982 (2)	0.0380 (10)	
N1	0.6627 (6)	0.8841 (3)	0.4684 (2)	0.0308 (11)	
N2	0.4137 (6)	0.8036 (3)	0.4390 (2)	0.0328 (12)	
N3	0.2967 (6)	0.8937 (3)	0.6757 (2)	0.0353 (12)	
H3N	0.3509	0.9185	0.6977	0.042*	
N4	0.0891 (6)	0.8240 (3)	0.6515 (2)	0.0376 (13)	
N5	0.8226 (5)	0.6114 (3)	0.0378 (2)	0.0297 (11)	
N6	1.0624 (6)	0.6873 (3)	0.0749 (2)	0.0302 (11)	
N7	1.2030 (6)	0.6180 (3)	−0.1701 (2)	0.0344 (12)	
H7N	1.1514	0.5949	−0.1939	0.041*	
N8	1.4042 (6)	0.6844 (3)	−0.1400 (2)	0.0366 (13)	
C1	0.7076 (7)	0.6401 (4)	0.4194 (3)	0.0352 (15)	
C2	0.7213 (8)	0.5488 (4)	0.4747 (3)	0.0472 (18)	
H2A	0.8212	0.5199	0.4619	0.057*	
H2B	0.7335	0.5566	0.5188	0.057*	
C3	0.5812 (9)	0.4880 (5)	0.4860 (4)	0.063 (2)	
H3A	0.5687	0.4816	0.4414	0.076*	
H3B	0.6112	0.4293	0.5176	0.076*	
C4	0.9721 (7)	0.8537 (4)	0.2988 (3)	0.0341 (14)	
C5	1.1306 (8)	0.9023 (6)	0.2622 (4)	0.070 (3)	
H5A	1.1023	0.9641	0.2366	0.084*	
H5B	1.1976	0.9032	0.2981	0.084*	
C6	1.2297 (10)	0.8697 (7)	0.2155 (4)	0.094 (4)	
H6A	1.1563	0.8615	0.1843	0.113*	
H6B	1.2648	0.8100	0.2434	0.113*	
C7	1.3780 (7)	0.9137 (4)	0.1705 (3)	0.0381 (15)	
H7A	1.3436	0.9539	0.1264	0.046*	
H7B	1.4478	0.8678	0.1601	0.046*	
C8	1.4824 (7)	0.9668 (4)	0.1990 (3)	0.0355 (14)	
H8A	1.5793	0.9881	0.1656	0.043*	
H8B	1.4193	1.0192	0.2011	0.043*	
C9	1.5386 (6)	0.9193 (4)	0.2688 (3)	0.0274 (13)	
C10	0.7904 (7)	0.9207 (4)	0.4835 (3)	0.0362 (15)	
H10	0.8879	0.9292	0.4534	0.043*	
C11	0.7861 (7)	0.9467 (4)	0.5417 (3)	0.0369 (15)	
H11	0.8792	0.9712	0.5502	0.044*	
C12	0.6438 (7)	0.9360 (4)	0.5862 (3)	0.0354 (14)	
H12	0.6385	0.9533	0.6253	0.042*	
C13	0.5047 (7)	0.8983 (4)	0.5718 (3)	0.0298 (13)	
C14	0.5209 (7)	0.8710 (4)	0.5128 (3)	0.0281 (13)	
C15	0.3495 (7)	0.8799 (4)	0.6141 (3)	0.0328 (14)	
C16	0.1398 (7)	0.8599 (4)	0.6950 (3)	0.0366 (15)	
C17	0.0455 (8)	0.8572 (4)	0.7621 (3)	0.0393 (16)	
C18	0.1196 (9)	0.8753 (4)	0.8142 (3)	0.0462 (17)	
H18	0.2295	0.8931	0.8064	0.055*	
C19	0.0269 (11)	0.8664 (5)	0.8771 (3)	0.057 (2)	
H19	0.0742	0.8794	0.9117	0.068*	
C20	−0.1355 (11)	0.8385 (5)	0.8894 (4)	0.061 (2)	
H20	−0.1946	0.8306	0.9328	0.073*	
C21	−0.2119 (9)	0.8221 (5)	0.8379 (4)	0.057 (2)	
H21	−0.3222	0.8051	0.8458	0.068*	
C22	−0.1186 (8)	0.8319 (5)	0.7742 (3)	0.0472 (18)	
H22	−0.1676	0.8212	0.7391	0.057*	
C23	0.2206 (7)	0.8376 (4)	0.5996 (3)	0.0293 (13)	
C24	0.2344 (7)	0.8094 (4)	0.5404 (3)	0.0335 (14)	
C25	0.3856 (7)	0.8275 (4)	0.4970 (3)	0.0290 (13)	
C26	0.1144 (7)	0.7657 (4)	0.5222 (3)	0.0394 (16)	
H26	0.0127	0.7527	0.5493	0.047*	
C27	0.1462 (8)	0.7416 (5)	0.4638 (3)	0.0464 (17)	
H27	0.0667	0.7118	0.4512	0.056*	
C28	0.2976 (7)	0.7620 (5)	0.4240 (3)	0.0407 (16)	
H28	0.3177	0.7453	0.3845	0.049*	
C29	0.7730 (7)	0.8424 (4)	0.1000 (3)	0.0294 (13)	
C30	0.7970 (8)	0.9353 (4)	0.0440 (3)	0.0417 (16)	
H30A	0.7550	0.9365	0.0013	0.050*	
H30B	0.7370	0.9799	0.0600	0.050*	
C31	0.9788 (8)	0.9563 (4)	0.0300 (3)	0.0414 (16)	
H31A	1.0380	0.9075	0.0193	0.050*	
H31B	1.0167	0.9593	0.0722	0.050*	
C32	0.5034 (7)	0.6247 (4)	0.2143 (3)	0.0348 (14)	
C33	0.3462 (7)	0.5743 (5)	0.2488 (3)	0.0474 (18)	
H33A	0.2811	0.5736	0.2122	0.057*	
H33B	0.3748	0.5126	0.2745	0.057*	
C34	0.2435 (9)	0.6090 (6)	0.2963 (4)	0.081 (3)	
H34A	0.2078	0.6683	0.2680	0.097*	
H34B	0.3162	0.6178	0.3275	0.097*	
C35	0.0948 (7)	0.5637 (5)	0.3412 (3)	0.0441 (17)	
H35A	0.0245	0.6090	0.3522	0.053*	
H35B	0.1296	0.5229	0.3851	0.053*	
C36	−0.0088 (7)	0.5111 (4)	0.3121 (3)	0.0402 (16)	
H36A	0.0548	0.4594	0.3087	0.048*	
H36B	−0.1056	0.4888	0.3453	0.048*	
C37	−0.0648 (6)	0.5623 (4)	0.2419 (3)	0.0302 (13)	
C38	0.6984 (7)	0.5767 (4)	0.0194 (3)	0.0381 (15)	
H38	0.5997	0.5662	0.0487	0.046*	
C39	0.7067 (7)	0.5549 (4)	−0.0414 (3)	0.0419 (16)	
H39	0.6156	0.5314	−0.0524	0.050*	
C40	0.8518 (7)	0.5691 (4)	−0.0841 (3)	0.0356 (15)	
H40	0.8612	0.5543	−0.1246	0.043*	
C41	0.9874 (7)	0.6060 (4)	−0.0672 (3)	0.0320 (14)	
C42	0.9655 (7)	0.6271 (4)	−0.0055 (3)	0.0294 (13)	
C43	1.1452 (7)	0.6283 (4)	−0.1076 (3)	0.0310 (14)	
C44	1.3593 (7)	0.6522 (4)	−0.1866 (3)	0.0346 (14)	
C45	1.4589 (7)	0.6573 (4)	−0.2526 (3)	0.0327 (14)	
C46	1.3919 (8)	0.6420 (4)	−0.3071 (3)	0.0413 (16)	
H46	1.2816	0.6260	−0.3022	0.050*	
C47	1.4920 (9)	0.6507 (4)	−0.3684 (3)	0.0444 (17)	
H47	1.4488	0.6396	−0.4046	0.053*	
C48	1.6558 (9)	0.6759 (4)	−0.3768 (3)	0.0481 (18)	
H48	1.7202	0.6832	−0.4191	0.058*	
C49	1.7243 (9)	0.6902 (4)	−0.3233 (3)	0.0490 (18)	
H49	1.8348	0.7059	−0.3285	0.059*	
C50	1.6238 (8)	0.6806 (4)	−0.2610 (3)	0.0407 (16)	
H50	1.6685	0.6900	−0.2244	0.049*	
C51	1.2684 (7)	0.6692 (4)	−0.0897 (3)	0.0315 (13)	
C52	1.2497 (6)	0.6902 (4)	−0.0268 (3)	0.0283 (13)	
C53	1.0969 (6)	0.6689 (4)	0.0145 (3)	0.0269 (13)	
C54	1.3660 (7)	0.7308 (4)	−0.0048 (3)	0.0398 (15)	
H54	1.4686	0.7455	−0.0309	0.048*	
C55	1.3313 (8)	0.7499 (4)	0.0560 (3)	0.0442 (17)	
H55	1.4090	0.7779	0.0712	0.053*	
C56	1.1771 (8)	0.7264 (4)	0.0940 (3)	0.0389 (16)	
H56	1.1540	0.7391	0.1352	0.047*	

### Atomic displacement parameters (Å^2^)

**Table:**

	*U*^11^	*U*^22^	*U*^33^	*U*^12^	*U*^13^	*U*^23^
Co1	0.0322 (4)	0.0377 (5)	0.0308 (4)	−0.0075 (4)	0.0032 (4)	−0.0184 (3)
Co2	0.0291 (4)	0.0251 (4)	0.0276 (4)	−0.0082 (3)	0.0040 (3)	−0.0120 (3)
Co3	0.0310 (4)	0.0309 (5)	0.0303 (4)	−0.0079 (3)	0.0022 (3)	−0.0154 (3)
O1	0.054 (3)	0.046 (3)	0.036 (2)	−0.004 (2)	0.001 (2)	−0.018 (2)
O2	0.049 (3)	0.032 (2)	0.032 (2)	−0.012 (2)	0.003 (2)	−0.0064 (17)
O3	0.042 (2)	0.053 (3)	0.044 (2)	−0.015 (2)	0.009 (2)	−0.028 (2)
O4	0.034 (2)	0.034 (2)	0.059 (3)	−0.0096 (19)	0.002 (2)	−0.024 (2)
O5	0.031 (2)	0.033 (2)	0.0305 (19)	0.0015 (18)	−0.0002 (17)	−0.0170 (17)
O6	0.044 (3)	0.041 (3)	0.045 (2)	−0.007 (2)	−0.002 (2)	−0.025 (2)
O7	0.054 (3)	0.029 (2)	0.028 (2)	−0.012 (2)	0.006 (2)	−0.0069 (16)
O8	0.049 (3)	0.034 (2)	0.034 (2)	−0.008 (2)	0.002 (2)	−0.0162 (18)
O9	0.029 (2)	0.040 (3)	0.050 (2)	−0.0129 (19)	0.0017 (19)	−0.027 (2)
O10	0.032 (2)	0.046 (3)	0.045 (2)	−0.008 (2)	0.009 (2)	−0.027 (2)
O11	0.033 (2)	0.027 (2)	0.036 (2)	0.0007 (18)	−0.0002 (18)	−0.0174 (17)
O12	0.042 (2)	0.033 (2)	0.046 (2)	−0.004 (2)	−0.002 (2)	−0.0233 (19)
N1	0.030 (3)	0.035 (3)	0.028 (2)	−0.009 (2)	−0.001 (2)	−0.012 (2)
N2	0.029 (3)	0.040 (3)	0.034 (2)	−0.008 (2)	−0.001 (2)	−0.019 (2)
N3	0.034 (3)	0.045 (3)	0.037 (3)	−0.005 (2)	−0.002 (2)	−0.026 (2)
N4	0.033 (3)	0.044 (3)	0.040 (3)	−0.010 (2)	0.004 (2)	−0.021 (2)
N5	0.027 (2)	0.028 (3)	0.033 (2)	−0.006 (2)	−0.001 (2)	−0.010 (2)
N6	0.032 (3)	0.031 (3)	0.030 (2)	−0.007 (2)	−0.006 (2)	−0.012 (2)
N7	0.039 (3)	0.040 (3)	0.031 (2)	−0.007 (2)	0.001 (2)	−0.021 (2)
N8	0.036 (3)	0.036 (3)	0.040 (3)	−0.010 (2)	0.007 (2)	−0.018 (2)
C1	0.028 (3)	0.040 (4)	0.032 (3)	−0.007 (3)	0.004 (3)	−0.007 (3)
C2	0.052 (4)	0.045 (4)	0.033 (3)	−0.007 (3)	−0.002 (3)	0.002 (3)
C3	0.057 (5)	0.038 (4)	0.076 (5)	−0.005 (4)	−0.002 (4)	0.003 (4)
C4	0.024 (3)	0.048 (4)	0.035 (3)	−0.008 (3)	−0.001 (3)	−0.020 (3)
C5	0.047 (4)	0.106 (7)	0.084 (5)	−0.037 (4)	0.029 (4)	−0.072 (5)
C6	0.067 (5)	0.163 (10)	0.084 (6)	−0.072 (6)	0.039 (5)	−0.088 (6)
C7	0.028 (3)	0.058 (4)	0.034 (3)	−0.005 (3)	−0.006 (3)	−0.021 (3)
C8	0.031 (3)	0.036 (4)	0.037 (3)	−0.005 (3)	−0.003 (3)	−0.009 (3)
C9	0.025 (3)	0.029 (3)	0.027 (3)	−0.007 (2)	0.007 (2)	−0.012 (2)
C10	0.025 (3)	0.048 (4)	0.043 (3)	−0.003 (3)	−0.004 (3)	−0.024 (3)
C11	0.029 (3)	0.042 (4)	0.045 (3)	−0.004 (3)	−0.006 (3)	−0.021 (3)
C12	0.045 (4)	0.029 (3)	0.037 (3)	−0.002 (3)	−0.011 (3)	−0.015 (3)
C13	0.036 (3)	0.024 (3)	0.032 (3)	−0.002 (3)	−0.003 (3)	−0.014 (2)
C14	0.030 (3)	0.027 (3)	0.031 (3)	−0.002 (2)	−0.004 (3)	−0.014 (2)
C15	0.034 (3)	0.036 (3)	0.032 (3)	−0.001 (3)	0.000 (3)	−0.018 (3)
C16	0.039 (3)	0.034 (4)	0.038 (3)	−0.003 (3)	0.008 (3)	−0.018 (3)
C17	0.050 (4)	0.034 (4)	0.034 (3)	−0.004 (3)	0.012 (3)	−0.017 (3)
C18	0.057 (4)	0.044 (4)	0.038 (3)	0.001 (3)	−0.003 (3)	−0.017 (3)
C19	0.099 (6)	0.052 (5)	0.027 (3)	0.021 (4)	−0.009 (4)	−0.023 (3)
C20	0.087 (6)	0.043 (4)	0.042 (4)	0.007 (4)	0.021 (4)	−0.012 (3)
C21	0.058 (5)	0.059 (5)	0.053 (4)	−0.011 (4)	0.018 (4)	−0.025 (4)
C22	0.049 (4)	0.059 (5)	0.038 (3)	−0.012 (3)	0.013 (3)	−0.026 (3)
C23	0.029 (3)	0.027 (3)	0.031 (3)	−0.001 (2)	0.002 (3)	−0.010 (2)
C24	0.030 (3)	0.041 (4)	0.031 (3)	0.002 (3)	−0.002 (3)	−0.016 (3)
C25	0.031 (3)	0.034 (3)	0.025 (3)	−0.003 (3)	−0.002 (3)	−0.013 (2)
C26	0.031 (3)	0.051 (4)	0.044 (3)	−0.014 (3)	−0.001 (3)	−0.026 (3)
C27	0.040 (4)	0.060 (5)	0.052 (4)	−0.014 (3)	−0.001 (3)	−0.035 (3)
C28	0.030 (3)	0.062 (5)	0.038 (3)	−0.011 (3)	0.000 (3)	−0.028 (3)
C29	0.025 (3)	0.028 (3)	0.029 (3)	0.003 (2)	0.002 (3)	−0.005 (2)
C30	0.050 (4)	0.036 (4)	0.037 (3)	−0.006 (3)	−0.001 (3)	−0.009 (3)
C31	0.052 (4)	0.034 (4)	0.031 (3)	−0.015 (3)	0.011 (3)	−0.006 (3)
C32	0.033 (3)	0.029 (3)	0.039 (3)	−0.005 (3)	−0.001 (3)	−0.009 (3)
C33	0.037 (4)	0.050 (4)	0.059 (4)	−0.021 (3)	0.013 (3)	−0.027 (3)
C34	0.059 (5)	0.122 (8)	0.095 (6)	−0.047 (5)	0.032 (5)	−0.085 (6)
C35	0.023 (3)	0.068 (5)	0.037 (3)	−0.001 (3)	0.002 (3)	−0.015 (3)
C36	0.031 (3)	0.039 (4)	0.044 (3)	−0.009 (3)	−0.005 (3)	−0.005 (3)
C37	0.018 (3)	0.034 (3)	0.038 (3)	−0.006 (2)	0.004 (3)	−0.014 (3)
C38	0.037 (3)	0.041 (4)	0.044 (3)	−0.011 (3)	0.003 (3)	−0.025 (3)
C39	0.035 (3)	0.053 (4)	0.045 (3)	−0.014 (3)	−0.001 (3)	−0.026 (3)
C40	0.035 (3)	0.036 (4)	0.040 (3)	−0.007 (3)	−0.002 (3)	−0.019 (3)
C41	0.032 (3)	0.031 (3)	0.036 (3)	−0.004 (3)	0.002 (3)	−0.017 (3)
C42	0.027 (3)	0.029 (3)	0.033 (3)	−0.007 (2)	0.001 (3)	−0.012 (2)
C43	0.035 (3)	0.029 (3)	0.030 (3)	−0.004 (3)	0.002 (3)	−0.013 (2)
C44	0.034 (3)	0.034 (3)	0.035 (3)	−0.009 (3)	0.005 (3)	−0.013 (3)
C45	0.041 (3)	0.026 (3)	0.032 (3)	−0.002 (3)	0.005 (3)	−0.014 (2)
C46	0.041 (4)	0.049 (4)	0.038 (3)	0.001 (3)	−0.003 (3)	−0.021 (3)
C47	0.063 (4)	0.036 (4)	0.032 (3)	0.003 (3)	0.002 (3)	−0.013 (3)
C48	0.059 (4)	0.041 (4)	0.040 (3)	0.005 (3)	0.013 (4)	−0.016 (3)
C49	0.049 (4)	0.048 (4)	0.050 (4)	−0.013 (3)	0.016 (3)	−0.022 (3)
C50	0.044 (4)	0.039 (4)	0.039 (3)	−0.007 (3)	0.007 (3)	−0.016 (3)
C51	0.030 (3)	0.028 (3)	0.039 (3)	−0.007 (2)	0.004 (3)	−0.016 (2)
C52	0.023 (3)	0.033 (3)	0.031 (3)	−0.005 (2)	−0.006 (2)	−0.012 (2)
C53	0.027 (3)	0.030 (3)	0.028 (3)	−0.005 (2)	0.001 (2)	−0.016 (2)
C54	0.032 (3)	0.051 (4)	0.039 (3)	−0.005 (3)	−0.004 (3)	−0.019 (3)
C55	0.046 (4)	0.051 (4)	0.045 (3)	−0.019 (3)	−0.004 (3)	−0.027 (3)
C56	0.047 (4)	0.047 (4)	0.032 (3)	−0.014 (3)	0.000 (3)	−0.024 (3)

### Geometric parameters (Å, °)

**Table:**

Co1—O1	2.016 (5)	C11—H11	0.9300
Co1—O3	2.022 (4)	C12—C13	1.412 (8)
Co1—O5^i^	2.156 (4)	C12—H12	0.9300
Co1—O6^i^	2.274 (5)	C13—C14	1.410 (7)
Co1—N1	2.098 (5)	C13—C15	1.426 (7)
Co1—N2	2.163 (4)	C14—C25	1.453 (8)
Co2—O2	2.088 (4)	C15—C23	1.387 (8)
Co2—O4	2.040 (4)	C16—C17	1.480 (7)
Co2—O7	2.061 (4)	C17—C22	1.383 (9)
Co2—O9	2.039 (4)	C17—C18	1.403 (9)
Co2—O5^i^	2.167 (4)	C18—C19	1.380 (8)
Co2—O11^ii^	2.176 (4)	C18—H18	0.9300
Co3—O8	2.030 (4)	C19—C20	1.382 (11)
Co3—O10	2.027 (4)	C19—H19	0.9300
Co3—O11^ii^	2.159 (4)	C20—C21	1.388 (11)
Co3—O12^ii^	2.264 (4)	C20—H20	0.9300
Co3—N5	2.097 (5)	C21—C22	1.393 (8)
Co3—N6	2.166 (4)	C21—H21	0.9300
O1—C1	1.271 (7)	C22—H22	0.9300
O2—C1	1.241 (7)	C23—C24	1.423 (8)
O3—C4	1.263 (7)	C24—C26	1.382 (8)
O4—C4	1.251 (7)	C24—C25	1.410 (7)
O5—C9	1.284 (6)	C26—C27	1.372 (8)
O5—Co1^ii^	2.156 (4)	C26—H26	0.9300
O5—Co2^ii^	2.167 (4)	C27—C28	1.381 (8)
O6—C9	1.252 (6)	C27—H27	0.9300
O6—Co1^ii^	2.274 (5)	C28—H28	0.9300
O7—C29	1.234 (6)	C29—C30	1.524 (8)
O8—C29	1.276 (7)	C30—C31	1.506 (9)
O9—C32	1.236 (7)	C30—H30A	0.9700
O10—C32	1.265 (7)	C30—H30B	0.9700
O11—C37	1.257 (7)	C31—C31^iv^	1.518 (10)
O11—Co3^i^	2.159 (4)	C31—H31A	0.9700
O11—Co2^i^	2.176 (4)	C31—H31B	0.9700
O12—C37	1.262 (7)	C32—C33	1.505 (7)
O12—Co3^i^	2.264 (4)	C33—C34	1.445 (8)
N1—C10	1.332 (7)	C33—H33A	0.9700
N1—C14	1.360 (6)	C33—H33B	0.9700
N2—C28	1.302 (7)	C34—C35	1.479 (8)
N2—C25	1.359 (7)	C34—H34A	0.9700
N3—C16	1.370 (7)	C34—H34B	0.9700
N3—C15	1.372 (7)	C35—C36	1.518 (9)
N3—H3N	0.8600	C35—H35A	0.9700
N4—C16	1.324 (8)	C35—H35B	0.9700
N4—C23	1.385 (6)	C36—C37	1.494 (8)
N5—C38	1.327 (7)	C36—H36A	0.9700
N5—C42	1.353 (6)	C36—H36B	0.9700
N6—C56	1.313 (7)	C38—C39	1.397 (8)
N6—C53	1.362 (6)	C38—H38	0.9300
N7—C44	1.366 (7)	C39—C40	1.363 (7)
N7—C43	1.371 (6)	C39—H39	0.9300
N7—H7N	0.8600	C40—C41	1.407 (8)
N8—C44	1.322 (7)	C40—H40	0.9300
N8—C51	1.388 (6)	C41—C42	1.408 (7)
C1—C2	1.504 (8)	C41—C43	1.431 (7)
C2—C3	1.475 (9)	C42—C53	1.454 (8)
C2—H2A	0.9700	C43—C51	1.373 (8)
C2—H2B	0.9700	C44—C45	1.470 (7)
C3—C3^iii^	1.467 (13)	C45—C50	1.385 (8)
C3—H3A	0.9700	C45—C46	1.396 (8)
C3—H3B	0.9700	C46—C47	1.380 (8)
C4—C5	1.512 (8)	C46—H46	0.9300
C5—C6	1.400 (9)	C47—C48	1.385 (10)
C5—H5A	0.9700	C47—H47	0.9300
C5—H5B	0.9700	C48—C49	1.377 (10)
C6—C7	1.472 (8)	C48—H48	0.9300
C6—H6A	0.9700	C49—C50	1.395 (7)
C6—H6B	0.9700	C49—H49	0.9300
C7—C8	1.519 (8)	C50—H50	0.9300
C7—H7A	0.9700	C51—C52	1.429 (7)
C7—H7B	0.9700	C52—C54	1.371 (8)
C8—C9	1.479 (8)	C52—C53	1.404 (7)
C8—H8A	0.9700	C54—C55	1.376 (8)
C8—H8B	0.9700	C54—H54	0.9300
C10—C11	1.387 (8)	C55—C56	1.391 (8)
C10—H10	0.9300	C55—H55	0.9300
C11—C12	1.366 (8)	C56—H56	0.9300
			
O1—Co1—O3	100.15 (19)	C23—C15—C13	123.3 (5)
O1—Co1—N1	97.09 (18)	N4—C16—N3	113.8 (5)
O3—Co1—N1	93.72 (17)	N4—C16—C17	124.4 (5)
O1—Co1—O5^i^	105.69 (17)	N3—C16—C17	121.6 (6)
O3—Co1—O5^i^	94.34 (15)	C22—C17—C18	119.8 (5)
N1—Co1—O5^i^	154.05 (18)	C22—C17—C16	118.2 (6)
O1—Co1—N2	86.74 (18)	C18—C17—C16	122.0 (6)
O3—Co1—N2	169.85 (18)	C19—C18—C17	119.0 (7)
N1—Co1—N2	77.94 (17)	C19—C18—H18	120.5
O5^i^—Co1—N2	90.92 (16)	C17—C18—H18	120.5
O1—Co1—O6^i^	163.61 (17)	C18—C19—C20	120.7 (7)
O3—Co1—O6^i^	87.28 (17)	C18—C19—H19	119.6
N1—Co1—O6^i^	96.99 (17)	C20—C19—H19	119.6
O5^i^—Co1—O6^i^	58.86 (15)	C19—C20—C21	121.1 (6)
N2—Co1—O6^i^	88.02 (17)	C19—C20—H20	119.4
O9—Co2—O4	178.17 (15)	C21—C20—H20	119.4
O9—Co2—O7	93.16 (17)	C20—C21—C22	118.1 (7)
O4—Co2—O7	85.93 (17)	C20—C21—H21	121.0
O9—Co2—O2	88.27 (16)	C22—C21—H21	121.0
O4—Co2—O2	92.67 (17)	C17—C22—C21	121.3 (7)
O7—Co2—O2	178.13 (17)	C17—C22—H22	119.3
O9—Co2—O5^i^	91.48 (16)	C21—C22—H22	119.3
O4—Co2—O5^i^	90.08 (16)	N4—C23—C15	110.6 (5)
O7—Co2—O5^i^	87.92 (15)	N4—C23—C24	127.5 (5)
O2—Co2—O5^i^	90.85 (15)	C15—C23—C24	121.8 (5)
O9—Co2—O11^ii^	88.14 (16)	C26—C24—C25	117.5 (5)
O4—Co2—O11^ii^	90.27 (16)	C26—C24—C23	126.1 (5)
O7—Co2—O11^ii^	90.28 (15)	C25—C24—C23	116.5 (5)
O2—Co2—O11^ii^	90.96 (15)	N2—C25—C24	121.9 (5)
O5^i^—Co2—O11^ii^	178.13 (14)	N2—C25—C14	116.6 (4)
O10—Co3—O8	99.43 (17)	C24—C25—C14	121.5 (5)
O10—Co3—N5	96.08 (16)	C27—C26—C24	119.6 (5)
O8—Co3—N5	95.76 (17)	C27—C26—H26	120.2
O10—Co3—O11^ii^	93.23 (15)	C24—C26—H26	120.2
O8—Co3—O11^ii^	105.97 (16)	C26—C27—C28	119.3 (6)
N5—Co3—O11^ii^	154.59 (17)	C26—C27—H27	120.4
O10—Co3—N6	171.27 (17)	C28—C27—H27	120.4
O8—Co3—N6	86.79 (17)	N2—C28—C27	123.2 (5)
N5—Co3—N6	77.10 (17)	N2—C28—H28	118.4
O11^ii^—Co3—N6	90.90 (16)	C27—C28—H28	118.4
O10—Co3—O12^ii^	88.27 (16)	O7—C29—O8	126.8 (5)
O8—Co3—O12^ii^	163.45 (16)	O7—C29—C30	117.7 (5)
N5—Co3—O12^ii^	97.97 (17)	O8—C29—C30	115.5 (5)
O11^ii^—Co3—O12^ii^	58.68 (14)	C31—C30—C29	108.4 (5)
N6—Co3—O12^ii^	87.31 (16)	C31—C30—H30A	110.0
C1—O1—Co1	127.0 (4)	C29—C30—H30A	110.0
C1—O2—Co2	134.4 (4)	C31—C30—H30B	110.0
C4—O3—Co1	121.3 (4)	C29—C30—H30B	110.0
C4—O4—Co2	138.2 (4)	H30A—C30—H30B	108.4
C9—O5—Co1^ii^	93.5 (3)	C30—C31—C31^iv^	113.7 (7)
C9—O5—Co2^ii^	142.7 (3)	C30—C31—H31A	108.8
Co1^ii^—O5—Co2^ii^	105.31 (17)	C31^iv^—C31—H31A	108.8
C9—O6—Co1^ii^	89.0 (4)	C30—C31—H31B	108.8
C29—O7—Co2	136.7 (4)	C31^iv^—C31—H31B	108.8
C29—O8—Co3	123.5 (4)	H31A—C31—H31B	107.7
C32—O9—Co2	139.8 (4)	O9—C32—O10	125.1 (5)
C32—O10—Co3	121.4 (4)	O9—C32—C33	118.5 (5)
C37—O11—Co3^i^	93.6 (3)	O10—C32—C33	116.5 (5)
C37—O11—Co2^i^	144.3 (3)	C34—C33—C32	116.2 (6)
Co3^i^—O11—Co2^i^	106.05 (17)	C34—C33—H33A	108.2
C37—O12—Co3^i^	88.7 (4)	C32—C33—H33A	108.2
C10—N1—C14	118.1 (5)	C34—C33—H33B	108.2
C10—N1—Co1	126.7 (4)	C32—C33—H33B	108.2
C14—N1—Co1	115.1 (4)	H33A—C33—H33B	107.4
C28—N2—C25	118.6 (4)	C33—C34—C35	124.6 (6)
C28—N2—Co1	128.0 (4)	C33—C34—H34A	106.2
C25—N2—Co1	113.3 (4)	C35—C34—H34A	106.2
C16—N3—C15	105.7 (5)	C33—C34—H34B	106.2
C16—N3—H3N	127.1	C35—C34—H34B	106.2
C15—N3—H3N	127.1	H34A—C34—H34B	106.4
C16—N4—C23	103.7 (5)	C34—C35—C36	117.2 (5)
C38—N5—C42	117.6 (5)	C34—C35—H35A	108.0
C38—N5—Co3	125.9 (3)	C36—C35—H35A	108.0
C42—N5—Co3	116.5 (4)	C34—C35—H35B	108.0
C56—N6—C53	118.0 (4)	C36—C35—H35B	108.0
C56—N6—Co3	128.1 (4)	H35A—C35—H35B	107.2
C53—N6—Co3	113.8 (3)	C37—C36—C35	115.4 (5)
C44—N7—C43	105.9 (5)	C37—C36—H36A	108.4
C44—N7—H7N	127.0	C35—C36—H36A	108.4
C43—N7—H7N	127.0	C37—C36—H36B	108.4
C44—N8—C51	104.2 (5)	C35—C36—H36B	108.4
O2—C1—O1	125.6 (6)	H36A—C36—H36B	107.5
O2—C1—C2	117.4 (6)	O11—C37—O12	118.9 (5)
O1—C1—C2	117.0 (6)	O11—C37—C36	121.7 (5)
C3—C2—C1	115.6 (6)	O12—C37—C36	119.5 (6)
C3—C2—H2A	108.4	N5—C38—C39	124.1 (5)
C1—C2—H2A	108.4	N5—C38—H38	118.0
C3—C2—H2B	108.4	C39—C38—H38	118.0
C1—C2—H2B	108.4	C40—C39—C38	118.2 (6)
H2A—C2—H2B	107.4	C40—C39—H39	120.9
C3^iii^—C3—C2	118.6 (9)	C38—C39—H39	120.9
C3^iii^—C3—H3A	107.7	C39—C40—C41	120.1 (5)
C2—C3—H3A	107.7	C39—C40—H40	120.0
C3^iii^—C3—H3B	107.7	C41—C40—H40	120.0
C2—C3—H3B	107.7	C40—C41—C42	117.3 (5)
H3A—C3—H3B	107.1	C40—C41—C43	127.0 (5)
O4—C4—O3	125.3 (5)	C42—C41—C43	115.6 (5)
O4—C4—C5	117.9 (5)	N5—C42—C41	122.7 (5)
O3—C4—C5	116.8 (5)	N5—C42—C53	116.3 (5)
C6—C5—C4	118.8 (6)	C41—C42—C53	121.0 (4)
C6—C5—H5A	107.6	N7—C43—C51	106.7 (4)
C4—C5—H5A	107.6	N7—C43—C41	129.7 (5)
C6—C5—H5B	107.6	C51—C43—C41	123.6 (5)
C4—C5—H5B	107.6	N8—C44—N7	113.1 (5)
H5A—C5—H5B	107.0	N8—C44—C45	124.9 (5)
C5—C6—C7	125.7 (7)	N7—C44—C45	121.9 (5)
C5—C6—H6A	105.9	C50—C45—C46	119.5 (5)
C7—C6—H6A	105.9	C50—C45—C44	118.2 (6)
C5—C6—H6B	105.9	C46—C45—C44	122.3 (5)
C7—C6—H6B	105.9	C47—C46—C45	119.0 (6)
H6A—C6—H6B	106.2	C47—C46—H46	120.5
C6—C7—C8	117.0 (5)	C45—C46—H46	120.5
C6—C7—H7A	108.0	C46—C47—C48	121.1 (7)
C8—C7—H7A	108.0	C46—C47—H47	119.4
C6—C7—H7B	108.0	C48—C47—H47	119.4
C8—C7—H7B	108.0	C49—C48—C47	120.6 (6)
H7A—C7—H7B	107.3	C49—C48—H48	119.7
C9—C8—C7	117.0 (5)	C47—C48—H48	119.7
C9—C8—H8A	108.0	C48—C49—C50	118.4 (6)
C7—C8—H8A	108.0	C48—C49—H49	120.8
C9—C8—H8B	108.0	C50—C49—H49	120.8
C7—C8—H8B	108.0	C45—C50—C49	121.4 (6)
H8A—C8—H8B	107.3	C45—C50—H50	119.3
O6—C9—O5	118.5 (5)	C49—C50—H50	119.3
O6—C9—C8	119.6 (5)	C43—C51—N8	110.1 (5)
O5—C9—C8	121.8 (5)	C43—C51—C52	121.7 (4)
N1—C10—C11	123.6 (5)	N8—C51—C52	128.2 (5)
N1—C10—H10	118.2	C54—C52—C53	118.0 (5)
C11—C10—H10	118.2	C54—C52—C51	125.6 (5)
C12—C11—C10	119.3 (6)	C53—C52—C51	116.4 (5)
C12—C11—H11	120.3	N6—C53—C52	122.0 (5)
C10—C11—H11	120.3	N6—C53—C42	116.3 (4)
C11—C12—C13	119.0 (5)	C52—C53—C42	121.7 (5)
C11—C12—H12	120.5	C52—C54—C55	120.1 (5)
C13—C12—H12	120.5	C52—C54—H54	119.9
C14—C13—C12	118.1 (5)	C55—C54—H54	119.9
C14—C13—C15	115.8 (5)	C54—C55—C56	118.3 (6)
C12—C13—C15	125.9 (5)	C54—C55—H55	120.8
N1—C14—C13	121.8 (5)	C56—C55—H55	120.8
N1—C14—C25	117.1 (5)	N6—C56—C55	123.5 (5)
C13—C14—C25	121.1 (4)	N6—C56—H56	118.2
N3—C15—C23	106.2 (4)	C55—C56—H56	118.2
N3—C15—C13	130.5 (5)		
			
O3—Co1—O1—C1	−99.9 (5)	C22—C17—C18—C19	0.8 (10)
N1—Co1—O1—C1	165.0 (4)	C16—C17—C18—C19	−176.2 (6)
O5^i^—Co1—O1—C1	−2.5 (5)	C17—C18—C19—C20	1.2 (10)
N2—Co1—O1—C1	87.6 (5)	C18—C19—C20—C21	−2.6 (11)
O6^i^—Co1—O1—C1	16.0 (8)	C19—C20—C21—C22	2.0 (11)
O9—Co2—O2—C1	−150.8 (6)	C18—C17—C22—C21	−1.3 (10)
O4—Co2—O2—C1	30.8 (6)	C16—C17—C22—C21	175.7 (6)
O5^i^—Co2—O2—C1	−59.3 (6)	C20—C21—C22—C17	0.0 (11)
O11^ii^—Co2—O2—C1	121.1 (6)	C16—N4—C23—C15	1.4 (7)
O1—Co1—O3—C4	59.4 (5)	C16—N4—C23—C24	177.8 (6)
N1—Co1—O3—C4	157.3 (5)	N3—C15—C23—N4	−0.7 (7)
O5^i^—Co1—O3—C4	−47.4 (5)	C13—C15—C23—N4	176.5 (5)
N2—Co1—O3—C4	−168.4 (10)	N3—C15—C23—C24	−177.4 (5)
O6^i^—Co1—O3—C4	−105.9 (5)	C13—C15—C23—C24	−0.2 (9)
O7—Co2—O4—C4	74.8 (6)	N4—C23—C24—C26	2.8 (11)
O2—Co2—O4—C4	−103.9 (6)	C15—C23—C24—C26	178.9 (6)
O5^i^—Co2—O4—C4	−13.1 (6)	N4—C23—C24—C25	−176.6 (6)
O11^ii^—Co2—O4—C4	165.1 (6)	C15—C23—C24—C25	−0.6 (9)
O9—Co2—O7—C29	−33.4 (6)	C28—N2—C25—C24	−0.8 (9)
O4—Co2—O7—C29	145.1 (6)	Co1—N2—C25—C24	−178.0 (4)
O5^i^—Co2—O7—C29	−124.7 (6)	C28—N2—C25—C14	178.0 (6)
O11^ii^—Co2—O7—C29	54.8 (6)	Co1—N2—C25—C14	0.8 (6)
O10—Co3—O8—C29	104.6 (4)	C26—C24—C25—N2	0.4 (9)
N5—Co3—O8—C29	−158.2 (4)	C23—C24—C25—N2	179.9 (5)
O11^ii^—Co3—O8—C29	8.5 (4)	C26—C24—C25—C14	−178.3 (6)
N6—Co3—O8—C29	−81.6 (4)	C23—C24—C25—C14	1.2 (9)
O12^ii^—Co3—O8—C29	−12.3 (7)	N1—C14—C25—N2	0.2 (8)
O7—Co2—O9—C32	102.7 (6)	C13—C14—C25—N2	−179.9 (5)
O2—Co2—O9—C32	−78.5 (6)	N1—C14—C25—C24	178.9 (5)
O5^i^—Co2—O9—C32	−169.3 (6)	C13—C14—C25—C24	−1.1 (9)
O11^ii^—Co2—O9—C32	12.5 (6)	C25—C24—C26—C27	0.2 (10)
O8—Co3—O10—C32	−61.0 (5)	C23—C24—C26—C27	−179.2 (6)
N5—Co3—O10—C32	−157.9 (5)	C24—C26—C27—C28	−0.4 (11)
O11^ii^—Co3—O10—C32	45.8 (5)	C25—N2—C28—C27	0.6 (10)
O12^ii^—Co3—O10—C32	104.3 (5)	Co1—N2—C28—C27	177.3 (5)
O1—Co1—N1—C10	92.0 (5)	C26—C27—C28—N2	0.0 (11)
O3—Co1—N1—C10	−8.8 (5)	Co2—O7—C29—O8	−12.9 (10)
O5^i^—Co1—N1—C10	−116.6 (5)	Co2—O7—C29—C30	169.5 (4)
N2—Co1—N1—C10	177.1 (6)	Co3—O8—C29—O7	−26.5 (8)
O6^i^—Co1—N1—C10	−96.5 (5)	Co3—O8—C29—C30	151.0 (4)
O1—Co1—N1—C14	−84.0 (4)	O7—C29—C30—C31	76.3 (7)
O3—Co1—N1—C14	175.3 (4)	O8—C29—C30—C31	−101.5 (6)
O5^i^—Co1—N1—C14	67.4 (5)	C29—C30—C31—C31^iv^	174.6 (6)
N2—Co1—N1—C14	1.1 (4)	Co2—O9—C32—O10	−47.3 (10)
O6^i^—Co1—N1—C14	87.6 (4)	Co2—O9—C32—C33	132.5 (5)
O1—Co1—N2—C28	−79.9 (6)	Co3—O10—C32—O9	5.9 (9)
O3—Co1—N2—C28	147.0 (10)	Co3—O10—C32—C33	−173.9 (4)
N1—Co1—N2—C28	−177.9 (6)	O9—C32—C33—C34	3.2 (10)
O5^i^—Co1—N2—C28	25.7 (6)	O10—C32—C33—C34	−176.9 (7)
O6^i^—Co1—N2—C28	84.5 (6)	C32—C33—C34—C35	−172.7 (7)
O1—Co1—N2—C25	96.9 (4)	C33—C34—C35—C36	−34.2 (12)
O3—Co1—N2—C25	−36.2 (13)	C34—C35—C36—C37	−54.2 (9)
N1—Co1—N2—C25	−1.0 (4)	Co3^i^—O11—C37—O12	4.3 (5)
O5^i^—Co1—N2—C25	−157.4 (4)	Co2^i^—O11—C37—O12	128.6 (5)
O6^i^—Co1—N2—C25	−98.6 (4)	Co3^i^—O11—C37—C36	−174.7 (4)
O10—Co3—N5—C38	8.2 (5)	Co2^i^—O11—C37—C36	−50.5 (9)
O8—Co3—N5—C38	−91.9 (5)	Co3^i^—O12—C37—O11	−4.1 (4)
O11^ii^—Co3—N5—C38	119.1 (5)	Co3^i^—O12—C37—C36	175.0 (4)
N6—Co3—N5—C38	−177.3 (5)	C35—C36—C37—O11	−38.1 (7)
O12^ii^—Co3—N5—C38	97.3 (5)	C35—C36—C37—O12	142.9 (5)
O10—Co3—N5—C42	−174.3 (4)	C42—N5—C38—C39	0.5 (9)
O8—Co3—N5—C42	85.5 (4)	Co3—N5—C38—C39	177.9 (5)
O11^ii^—Co3—N5—C42	−63.5 (6)	N5—C38—C39—C40	0.8 (10)
N6—Co3—N5—C42	0.1 (4)	C38—C39—C40—C41	−1.0 (10)
O12^ii^—Co3—N5—C42	−85.2 (4)	C39—C40—C41—C42	−0.1 (9)
O8—Co3—N6—C56	81.9 (5)	C39—C40—C41—C43	−177.4 (6)
N5—Co3—N6—C56	178.6 (6)	C38—N5—C42—C41	−1.7 (9)
O11^ii^—Co3—N6—C56	−24.1 (5)	Co3—N5—C42—C41	−179.3 (4)
O12^ii^—Co3—N6—C56	−82.6 (5)	C38—N5—C42—C53	177.4 (5)
O8—Co3—N6—C53	−96.6 (4)	Co3—N5—C42—C53	−0.3 (7)
N5—Co3—N6—C53	0.0 (4)	C40—C41—C42—N5	1.5 (9)
O11^ii^—Co3—N6—C53	157.4 (4)	C43—C41—C42—N5	179.1 (5)
O12^ii^—Co3—N6—C53	98.9 (4)	C40—C41—C42—C53	−177.5 (6)
Co2—O2—C1—O1	23.4 (9)	C43—C41—C42—C53	0.1 (8)
Co2—O2—C1—C2	−155.7 (4)	C44—N7—C43—C51	0.8 (6)
Co1—O1—C1—O2	15.5 (8)	C44—N7—C43—C41	178.3 (6)
Co1—O1—C1—C2	−165.4 (4)	C40—C41—C43—N7	−1.3 (11)
O2—C1—C2—C3	−56.1 (8)	C42—C41—C43—N7	−178.6 (6)
O1—C1—C2—C3	124.7 (7)	C40—C41—C43—C51	175.9 (6)
C1—C2—C3—C3^iii^	−65.4 (12)	C42—C41—C43—C51	−1.4 (9)
Co2—O4—C4—O3	46.2 (10)	C51—N8—C44—N7	0.6 (7)
Co2—O4—C4—C5	−132.0 (6)	C51—N8—C44—C45	176.3 (6)
Co1—O3—C4—O4	−4.4 (9)	C43—N7—C44—N8	−0.9 (7)
Co1—O3—C4—C5	173.9 (5)	C43—N7—C44—C45	−176.7 (5)
O4—C4—C5—C6	−0.9 (12)	N8—C44—C45—C50	13.8 (9)
O3—C4—C5—C6	−179.3 (8)	N7—C44—C45—C50	−170.8 (6)
C4—C5—C6—C7	173.5 (8)	N8—C44—C45—C46	−164.5 (6)
C5—C6—C7—C8	33.0 (13)	N7—C44—C45—C46	10.8 (9)
C6—C7—C8—C9	53.8 (9)	C50—C45—C46—C47	−0.3 (9)
Co1^ii^—O6—C9—O5	3.7 (4)	C44—C45—C46—C47	178.0 (6)
Co1^ii^—O6—C9—C8	−175.0 (4)	C45—C46—C47—C48	−1.1 (10)
Co1^ii^—O5—C9—O6	−3.9 (5)	C46—C47—C48—C49	1.9 (10)
Co2^ii^—O5—C9—O6	−125.0 (5)	C47—C48—C49—C50	−1.3 (10)
Co1^ii^—O5—C9—C8	174.8 (4)	C46—C45—C50—C49	0.9 (10)
Co2^ii^—O5—C9—C8	53.7 (8)	C44—C45—C50—C49	−177.5 (6)
C7—C8—C9—O6	−145.8 (5)	C48—C49—C50—C45	−0.1 (10)
C7—C8—C9—O5	35.5 (7)	N7—C43—C51—N8	−0.4 (7)
C14—N1—C10—C11	−0.6 (9)	C41—C43—C51—N8	−178.2 (5)
Co1—N1—C10—C11	−176.4 (5)	N7—C43—C51—C52	−180.0 (5)
N1—C10—C11—C12	−0.8 (10)	C41—C43—C51—C52	2.3 (10)
C10—C11—C12—C13	0.2 (9)	C44—N8—C51—C43	−0.1 (7)
C11—C12—C13—C14	1.7 (9)	C44—N8—C51—C52	179.4 (6)
C11—C12—C13—C15	177.8 (6)	C43—C51—C52—C54	179.9 (6)
C10—N1—C14—C13	2.6 (9)	N8—C51—C52—C54	0.5 (10)
Co1—N1—C14—C13	178.9 (4)	C43—C51—C52—C53	−1.7 (9)
C10—N1—C14—C25	−177.4 (5)	N8—C51—C52—C53	178.8 (6)
Co1—N1—C14—C25	−1.1 (7)	C56—N6—C53—C52	0.2 (9)
C12—C13—C14—N1	−3.2 (9)	Co3—N6—C53—C52	178.9 (4)
C15—C13—C14—N1	−179.7 (5)	C56—N6—C53—C42	−178.9 (6)
C12—C13—C14—C25	176.8 (5)	Co3—N6—C53—C42	−0.2 (6)
C15—C13—C14—C25	0.3 (8)	C54—C52—C53—N6	−0.1 (9)
C16—N3—C15—C23	−0.3 (7)	C51—C52—C53—N6	−178.5 (5)
C16—N3—C15—C13	−177.2 (6)	C54—C52—C53—C42	179.0 (6)
C14—C13—C15—N3	176.7 (6)	C51—C52—C53—C42	0.5 (8)
C12—C13—C15—N3	0.6 (11)	N5—C42—C53—N6	0.4 (8)
C14—C13—C15—C23	0.3 (9)	C41—C42—C53—N6	179.4 (5)
C12—C13—C15—C23	−175.9 (6)	N5—C42—C53—C52	−178.7 (5)
C23—N4—C16—N3	−1.6 (7)	C41—C42—C53—C52	0.3 (9)
C23—N4—C16—C17	−176.1 (6)	C53—C52—C54—C55	−0.3 (10)
C15—N3—C16—N4	1.2 (7)	C51—C52—C54—C55	178.1 (6)
C15—N3—C16—C17	175.9 (6)	C52—C54—C55—C56	0.5 (10)
N4—C16—C17—C22	−13.3 (10)	C53—N6—C56—C55	0.0 (10)
N3—C16—C17—C22	172.6 (6)	Co3—N6—C56—C55	−178.4 (5)
N4—C16—C17—C18	163.6 (7)	C54—C55—C56—N6	−0.3 (11)
N3—C16—C17—C18	−10.5 (10)		

Symmetry codes: (i) *x*−1, *y*, *z*; (ii) *x*+1, *y*, *z*; (iii) −*x*+1, −*y*+1, −*z*+1; (iv) −*x*+2, −*y*+2, −*z*.

### Hydrogen-bond geometry (Å, °)

**Table:**

*D*—H···*A*	*D*—H	H···*A*	*D*···*A*	*D*—H···*A*
N3—H3N···O6^v^	0.86	2.05	2.785 (6)	143
N7—H7N···O12^vi^	0.86	2.05	2.795 (6)	144

Symmetry codes: (v) −*x*+2, −*y*+2, −*z*+1; (vi) −*x*+1, −*y*+1, −*z*.
